# Description of Joint Alterations Observed in a Family Carrying p.Asn453Ser *COMP* Variant: Clinical Phenotypes, In Silico Prediction of Functional Impact on *COMP* Protein and Stability, and Review of the Literature

**DOI:** 10.3390/biom11101460

**Published:** 2021-10-05

**Authors:** Quitterie Rochoux, Jana Sopkova-de Oliveira Santos, Christian Marcelli, Anne Rovelet-Lecrux, Virginie Chevallier, Jean-Jacques Dutheil, Sylvain Leclercq, Karim Boumédiene, Catherine Baugé, Juliette Aury-Landas

**Affiliations:** 1 EA7451 BioConnecT, Normandie Université, UNICAEN, 14032 Caen, France; rochoux-q@chu-caen.fr (Q.R.); sylvainleclercq60@gmail.com (S.L.); karim.boumediene@unicaen.fr (K.B.); catherine.bauge@unicaen.fr (C.B.); 2 Service de Rhumatologie, CHU de Caen, 14000 Caen, France; marcelli-c@chu-caen.fr; 3 EA4258 CERMN, Normandie Université, UNICAEN, 14032 Caen, France; jana.sopkova@unicaen.fr; 4 Department of Genetics and CNR-MAJ, Normandy Center for Genomic and Personalized Medicine, Normandie Université, UNIROUEN, Inserm U1245 and Rouen University Hospital, 76000 Rouen, France; anne.roveletlecrux@univ-rouen.fr; 5 Délégation de la Recherche Clinique et de l’Innovation, CHU de Caen, 14000 Caen, France; chevallier-v@chu-caen.fr (V.C.); dutheil-jj@chu-caen.fr (J.-J.D.); 6 Service de Chirurgie Orthopédique, Clinique Saint-Martin, 14000 Caen, France

**Keywords:** mutation, genetics, arthritis, osteoarthritis, *COMP*, dysplasia

## Abstract

The role of genetics in the development of osteoarthritis is well established but the molecular bases are not fully understood. Here, we describe a family carrying a germline mutation in *COMP* (*Cartilage Oligomeric Matrix Protein*) associated with three distinct phenotypes. The index case was enrolled for a familial form of idiopathic early-onset osteoarthritis. By screening potential causal genes for osteoarthritis, we identified a heterozygous missense mutation of *COMP* (c.1358C>T, p.Asn453Ser), absent from genome databases, located on a highly conserved residue and predicted to be deleterious. Molecular dynamics simulation suggests that the mutation destabilizes the overall COMP protein structure and consequently the calcium releases from neighboring calcium binding sites. This mutation was once reported in the literature as causal for severe multiple epiphyseal dysplasia (MED). However, no sign of dysplasia was present in the index case. The mutation was also identified in one of her brothers diagnosed with MED and secondary osteoarthritis, and in her sister affected by an atypical syndrome including peripheral inflammatory arthritis of unknown cause, without osteoarthritis nor dysplasia. This article suggests that this mutation of *COMP* is not only causal for idiopathic early-onset osteoarthritis or severe MED, but can also be associated to a broad phenotypic variability with always joint alterations.

## 1. Introduction

Osteoarthritis (OA) is the most common worldwide joint disease [1]. It is a complex disease, caused by a combination of three main risk factors: genetics, environmental factors, and age [2]. The first study demonstrating the importance of genetic factors in OA was carried out by Stecher in 1941 [3]. Since then, numerous studies have demonstrated that the genetic influence depends on the localization of the disease, and is comprised between 35% and 65% [4,5,6]. A large number of studies support the theory of a polygenic inheritance, as opposed to defect in a single gene [4]. However, rare OA forms, which are characterized by the early onset of the disease, may represent Mendelian diseases with an autosomal dominant inheritance pattern. To date, mutations in 13 genes have been reported to be potentially causal for OA [5,7]. These genes encode components of the extracellular matrix (*COL2A1*, *COL9A1*, *COL9A2*, *COL9A3*, *COL11A1*, *COL11A2*, *ACAN, COMP* and *MATN3*), the transforming growth factor b pathway (*SMAD3*), vesicular transport (*TRAPPC2*) or bone remodeling proteins (*TNFRSF11B* and *TUFT1*). However, these genes do not explain all the genetic forms of the disease, and some of them are not fully validated. Thus, the identification of disease-causing genes for OA remains important to unravel the etiology and pathogenesis of this disease in order to highlight potential targets for curative treatment. Indeed, the treatment of OA remains a problem because none of the current therapies can promote the regeneration of degenerated tissues. Regenerative medicine is an innovative approach, which is being evaluated such as the use of mesenchymal stem cells in combination with bio-scaffolds [8,9,10].

Here, we describe a family harboring a germline heterozygous mutation of the *COMP* gene (c.1358C>T, p.Asn453Ser) associated with three distinct phenotypes, including one atypical syndrome reported for the first time. This highlights the extreme clinical variability of this mutation even within the same family.

## 2. Subjects and Methods

### 2.1. Subjects

This study was approved by the local Ethics Committee, named “Comité de Protection des Personnes Nord Ouest III” (ID RCB 2013-A00211-44) and CNIL (905.515), and deposited at clinical trial website as Exorhum Project (NCT01999166, registered in 2013). All patients signed an informed consent before inclusion in the study.

A patient with early-onset OA from a French family was identified and recruited by rheumatologists at the Centre Hospitalier Universitaire of Caen, and two of her family members were also enrolled. Clinical presentations including medical history, physical examination, and radiography as well as peripheral blood were collected from all enrolled subjects. Clinical data were collected and reviewed by rheumatologists on the basis of X-rays, medical reports or clinical examinations (III.1, III.2, III.3, III.4 and IV.9) or relatives’ narration.

### 2.2. Genetic Analyses

Genomic DNA was extracted from peripheral blood using FlexiGene DNA kit (Qiagen, Courtaboeuf, France) according to the manufacturer’s instructions, and quantified on Multiskan GO spectrophotometer (ThermoFisher Scientific, Courtaboeuf, France). DNA integrity was checked on 1.2% agarose gel. Whole-exome sequencing was performed by Integragen Genomics (Evry, France) on HiSeq4000 (Illumina, Evry, France) after exome capture using SureSelect XT Human All Exon V5-UTR (Agilent technologies, Les Ulis, France). Sequencing reads alignment to the human reference genome hg19/GRCh37 and variant calling were processed using the CASAVA v1.8 pipeline (Illumina). Variants were annotated using Annovar software [11]. Filtering was performed following strict criteria and consisted of removing any low confidence variants (not passing the Variant Quality Score Recalibration from GATK tools) and excluding variants with a minor allele frequency (MAF) > 0.001 reported in Exome Aggregation Consortium (ExAC) or the Genome Aggregation Database (gnomAD v2.1.1). Variants present in the Integragen reference database with a MAF > 0.01 were excluded as they must correspond to false positives related to the technology. For this study, we screened known pathogenic genes of OA (*COL2A1*, *COL9A1*, *COL9A2*, *COL9A3*, *COL11A1*, *COL11A2*, *ACAN, COMP, MATN3*, SMAD3, *TRAPPC2, TNFRSF11B* and *TUFT1)*. Mutation validation and co-segregation analysis were performed by Sanger sequencing.

### 2.3. In Silico Variant Pathogenicity Prediction Analysis

To predict the potential pathogenicity of genetic variants, in silico prediction analysis was performed according to the American College of Medical Genetics and Genomics guidelines [12].

To evaluate the evolutionary conservation, twelve FASTA sequences of *COMP* homologs were obtained from ”homologene” and “protein” databases of National Center for Biotechnology Information (NCBI), U.S. National Library of Medicine: NP_000086.2 for Homo sapiens, NP_001092035.1 for Pan troglodytes, XP_014978841.2 for Macaca mulatta, XP_533869.2 for Canis lupus familiaris, NP_001159989.2 for Bos Taurus, NP_057894.2 for Mus musculus, NP_036966.1 for Rattus norvegicus, for XP_418238.2 for Gallus gallus, XP_012821130.2 for Xenopus tropicalis, NP_001313279.1 for Danio rerio, NP_523495.2 for Drosophila melanogaster, and XP_308033.5 for Anopheles gambiae. Multiple sequence alignment of COMP protein sequences from these different species was performed by Clustal Omega program [13].

Four in silico prediction tools were used to evaluate variant pathogenicity: MutationTaster [14], PolyPhen-2 HumDiv [15], Sorting Intolerant From Tolerant (SIFT) [16], and Combined Annotation Dependent Depletion (CADD) [17]. Conserved domains database (CDD) [18] was used to identify structural domains and Ca^2+^ binding sites present on the COMP protein (NP_000086.2).

### 2.4. Molecular Dynamics Simulations

The crystal structure of signature domain of COMP (PDB ID: 3FBY) was used as a starting structure for all molecular dynamics simulations [19]. Two molecular dynamics simulations of 100 ns each were produced: a wild type (WT) and N453S one on which the Asn453 was mutated to a Ser using PyMOL mutagenesis tool (The PyMOL Molecular Graphics System, Version 2.0, Schrödinger, L., & DeLano, W. (2020). *PyMOL*. Retrieved from http://www.pymol.org/pymol, accessed on 24 July 2021). Both simulations were carried out using NAMD 2.12 [20] with the all-atom Chemistry at HARvard Macromolecular Mechanics (CHARMM) 36 m force field [21,22]. To simulate the aqueous solvent environment, each system was surrounded by a rectangular box of TIP3P water molecules [23] and 0.15 M of KCl was added to the system using CHARMmGUIsolvator [24]. The chosen box size ensured, for each complex, that the simulated complex was at a minimum distance of 10 Å from the edge. Periodic boundary conditions were applied to the systems using the ‘IMAGE’ algorithm. Van der Waals interactions were truncated using a force switching function between 10 and 12 Å and the particle mesh Ewald [25] was used to calculate long-range electrostatic interactions. The ‘SHAKE’ algorithm was applied to restrain all bonds involving hydrogen atoms [26]. The vacuum dielectric constant was used during all calculations. Firstly, the systems underwent energy minimization in 10,000 steps. Next, the minimized systems were heated to 303.15 K and the dynamics were temperature-equilibrated during 50 ps via heating reassignment under constant atoms’ number, constant volume, and constant temperature - NVT conditions. Finally, the systems ran freely for 100 ns under constant atoms’ number, pressure, and temperature (NPT conditions) with a time step of 2 fs. Langevin dynamics with a damping coefficient of 1 ps^−1^ were used to maintain the system temperature and the Nosé-Hover Langevin piston method was used to control the pressure at 1 atm. Production trajectories were saved every 100 ps and subsequent analyses were performed using the CHARMM program version c40b2 [27].

## 3. Results

### 3.1. Clinical Presentation

**Figure 1:**
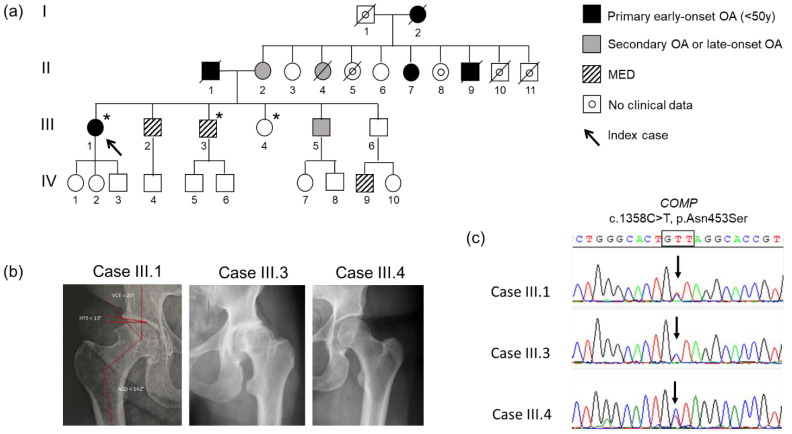
Pedigree, radiographic findings and *COMP* mutation co-segregation in the family. (**a**) Pedigree of the family. Asterisk represents individuals genotyped for p.Asn453Ser *COMP* mutation. Black color filled symbols indicate patients with idiopathic early-onset osteoarthritis, gray color filled symbols indicate patients with dysplasia, and uncolored symbols indicate unaffected individuals. The arrow indicates the index case. (**b**) Hip joint radiographic findings of the 3 genotyped cases. Right hip joint of the index case (III.1) at the age of 50 years shows signs of hip osteoarthritis with superior medial narrowing of the coxofemoral joint space, subchondral condensation, and pericapital osteophytic production (Kellgren and Lawrence score of 3), without signs of dysplasia. Hip joint of his brother (III.3) shows dysplasia. No dysplasia nor osteoarthritis were shown on hip joint of her sister (III.4). (**c**) Electropherogram obtained by Sanger sequencing of germline DNA from index case affected by early onset osteoarthritis without dysplasia (III.1), her brother affected by dysplasia (III.3), and her unaffected sister (III.4). The arrows indicate the presence of c.1358A>G mutation of *COMP* in the 3 cases.

A four-generation French family with autosomal dominant inheritance of early-onset hip OA (Figure 1a) was identified. Three members from this family (members III.1, III.3 and III.4) were recruited. Phenotypes of the index case and her relatives include joint pain, early-onset hip OA necessitating bilateral hip prosthesis (II.10, III.1, III.2 and III.3), and mild short stature (around 155–160 cm on average).

The index case (III.1) is a 51-year-old woman diagnosed with severe early-onset OA of the hip, which necessitated a total bilateral hip prosthesis at the ages of 47 and 51 years old. Bilateral hip pain complaints started at the age of 25 years old. No sign of dysplasia was present on hips, knees, shoulders, elbows or spine X-rays (Figure 1b). Outside the OA, she did not complain of any other pathology. She measured 157 cm and 68 kg. No evident environmental causes of OA (congenital skeletal abnormalities, acquired bone diseases, chronic inflammatory rheumatism, metabolic diseases, obesity and traumatisms) could be identified. Thus, her clinical presentation is strongly evocative of a genetic form of OA (familial history, precocity, severity and absence of evident environmental cause).

Besides a family history of idiopathic early-onset hip OA, two first-degree relatives of the index case (III.2 and III.3) were diagnosed with multiple epiphyseal dysplasia (MED), as shown on X-rays for III.3 (Figure 1b). The brother III.6 had no symptoms but the diagnosis of MED was made recently for his 10-year-old son. Moreover, three first-degree relatives of the index case presented atypical phenotypes.

The brother III.5 presented fractures of the femoral neck at 13 years and 35 years, respectively, after moderate trauma, which is surprising and unusual in young people with no medical history. The brother III.3 presented a spontaneous osteonecrosis of the medial femoral condyle at 45 years, without obvious etiology, having required a valgisation osteotomy. This spontaneous osteonecrosis could indicate a local anomaly, as well as the femoral neck fracture in these two men.

The sister (III.4) presented a syndromic phenotype, including dwarfism (138 cm), mild facial dysmorphic features, brachydactyly without clubbing, moderate intellectual disability and, more recently, a progressive development of a sensorineural hearing loss, a premature ovarian insufficiency and a peripheral inflammatory arthritis. Regarding rheumatism, it started at the age of 39. Pain predominated in the hands, knees with swelling and shoulders. The wrist MRI showed inflammatory extensor synovitis and flexor and extensor tenosynovitis. The knee puncture had found a sterile but inflammatory fluid (6000 elements), without crystals. To explore the rheumatism, the biological analyzes did not show any abnormality, in particular auto-immune analyzes (including Rheumatoid factor and antibodies to cyclic citrullinated peptide, CCP). The radiographs did not find any anomalies and, in particular, no chondrodysplasia. Bone scintigraphy found moderate fixations of the knees, carpus and right ulno-carpal joint, right hip and last lumbar vertebra, but aspecific, described as rather of degenerative origin. The recent lumbar spine and sacro-iliac MRI were normal. As such, she presented with atypical rheumatism, seronegative, in a context of syndromic picture. She benefited from an opinion from geneticists who eliminated the diagnosis of pseudoachondroplasia, known to be associated with *COMP* mutation.

The father (II.1) developed early-onset OA, necessitating bilateral hip prosthesis before the age of 50.

On the maternal side, the mother (II.2), aged 70, presents rather late pain with in particular a coxarthrosis awaiting total hip arthroplasty. Among her six sisters, two were carrying total hip prosthesis. Among her three brothers, one complains of coxalgia that started around the age of 14, requiring bilateral hip prosthesis at 49 years old. He had six children, including four sons, all having coxalgia.

### 3.2. Genetic Analysis and Co-Segregation

**Figure 2:**
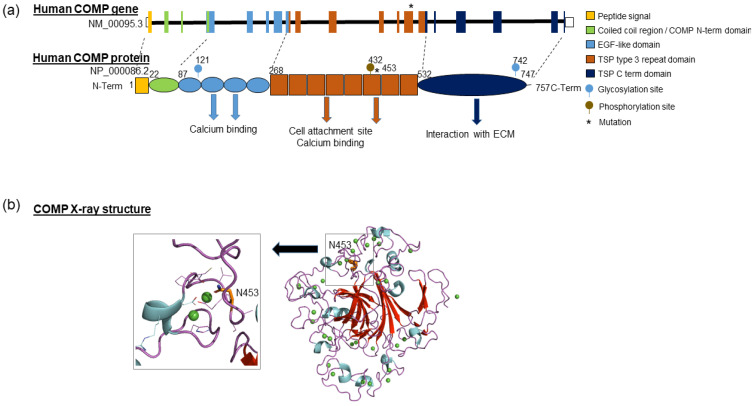
Schematic representation of *COMP* gene and protein. (**a**) Human *COMP* gene is long of 9532bp, located in chromosome 19 and composed of 19 exons. It encodes a pentameric protein of 524 kDa in which the monomers are joined together by a coiled coil domain in the N terminus. Each N-terminal domain is followed by four EGF repeat domains, eight thrombospondin (TSP) type III domains, and a C-terminal globular domain. The EGF repeat and thrombospondin type III domains are able to bind calcium ions, while the C-terminal domain is involved in interactions with other proteins in the extracellular matrix (collagens, fibronectin, aggrecan). (**b**) Ribbon representation of X-ray structure of COMP protein: β-sheet are colored in red, α-helices in cyan and the coils/loops in pink. Ca^2+^ ions are schematized as green spheres. Left panel: Detailed view of Ca^2+^ binding site with N453.

In order to identify the molecular basis underlying the early-onset OA form of the index case, genes already described to be causal for early-onset OA were screened by whole exome sequencing. Only a rare heterozygous variant c.1358A>G (NM_000095.2) was identified within exon 13 of the *COMP* gene encoding Cartilage Oligomeric Matrix Protein, and confirmed by Sanger sequencing (Figure 1c). This missense variant results in the substitution of the asparagine in position 453 by a serine (NP_000086.2:p.Asn453Ser) in a thrombospondin-type 3 repeat, corresponding to a calcium-binding domain of the COMP protein (Figure 2a).

One of her brothers (III.3) who underwent bilateral total hip replacement at 40 and 41 years old was found to be also heterozygous for the *COMP* mutation. Surprisingly, this mutation was also present in the genomic DNA of her sister (III.4, 43 years) who was not affected by OA but had a peripheral inflammatory arthritis (Figure 1c).

### 3.3. In Silico Prediction of Variation Pathogenicity

**Figure 3:**
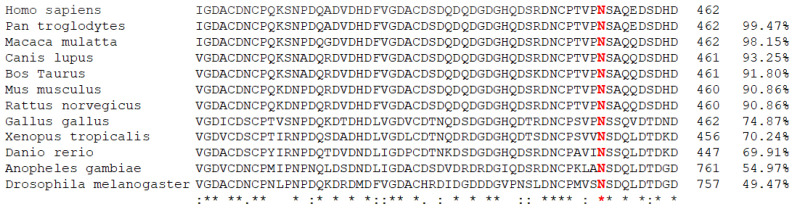
Evolutionary conservation of COMP protein around the position 453 (asparagine). Multiple sequence alignment of COMP protein sequences from different species was performed by Clustal Omega program. Residue in red (N) corresponds to the asparagine located at position 453 in the human protein (NP_000086.2). Percentage corresponds to global identity compared to the human COMP protein. Asterisk represents individuals genotyped for p.Asn453Ser COMP mutation.

The mutated residue (Asparagine at position 453) is highly conserved in different species: it is present at least until Bilateria lineage (Figure 3). The variation p.Asn453Ser is considered as deleterious by four in silico prediction tools, with the following annotation: “deleterious” by SIFT (score = 0), “probably damaging” by PolyPhen2 HumDiv (score = 0.99), “disease causing” by MutationTaster (score = 1), and “pathogenic” by CADD (Phred score = 23.8). Interestingly, this variant is totally absent from the general population (gnomAD). It is predicted as “pathogenic” in Clinvar databases (Variation ID 9190), and has been reported in a patient with severe MED [28]. However, no obvious sign of dysplasia was detected in the index case, nor in her sister (III.4) although some related present MED phenotype (namely III.3 case who has also the variation in the *COMP* gene).

### 3.4. Structural Analysis and Molecular Dynamics Simulation

**Figure 4:**
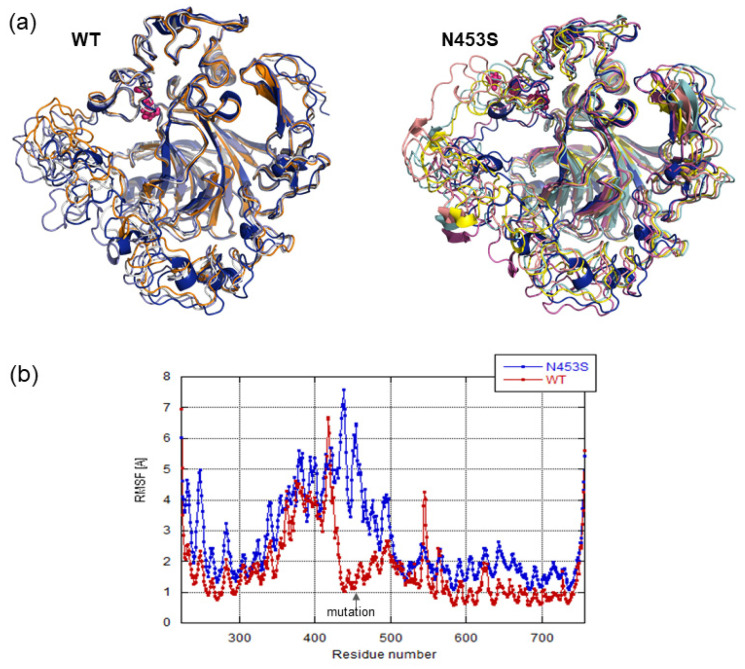
Molecular dynamics simulation. (**a**) Ribbon representation of the superposition of representative structures from the WT and N453S dynamics. (**b**) The root mean squared fluctuations calculated on backbone atoms per COMP residue for WT and N453S dynamics simulations.

Using the conserved domains database, we could observe that the variation induced a mutation of a residue essential for Ca^2+^ binding (Figure 2b), suggesting that the variation significantly affects Ca^2+^ binding, and consequently protein folding. As such, we checked calcium binding ability as well as whole protein stability through molecular dynamics simulations on the WT protein and mutated one (N453S). The visualization of molecular dynamics trajectories allowed us to observe that the COMP beta sheet core stayed stable in both simulations and that the loops binding the calcium ions fluctuated highly (Figure 4a). This was confirmed by the root mean squared fluctuations calculated on backbone atoms from both simulations (Figure 4b). In the majority, the calcium ions remained in their binding sites and the loops forming these binding sites changed the position during the dynamic. The detected mutation is situated in one loop binding calcium ion and the Asn453 coordinates directly the binding of calcium ion (Figure 2a). In the WT simulation we observed that the fluctuations occurring in this calcium binding loops region did not affect the calcium ion binding. In the N453S dynamics we observed that the mutation did not disturb the calcium binding in this site, and Ser453 coordinated the calcium ion in this site during all simulations. However, we observed a greater amplitude of the fluctuations in the region surrounding the mutation site (Figure 4a), which affected mainly the loops binding the calcium ions situated before the mutated loop. The neighboring loops’ restructuration during the simulation induced a release of calcium ion from the neighboring binding site in N453S dynamics. Looking at the simulations’ results, we can conclude that the Asn453Ser mutation, while not influencing directly the target calcium binding site, induces an important mobility in its environment and therefore destabilizes the COMP protein and consequently other calcium binding sites.

## 4. Discussion

Here, we describe an extreme clinical variability including atypical phenotypes in a family with a p.Asn453Ser COMP mutation. Cartilage oligomeric matrix protein gene, also known as thrombospondin 5, encodes a 524 kDa pentameric extracellular matrix glycoprotein member of the thrombospondin family of calcium-binding proteins. It interacts with multiple extracellular matrices, catalyzes collagen assembly and promotes the formation of well-defined fibrils.

Originally described in cartilage, COMP has also been identified in numerous tissues, such as adipose tissue, skeletal muscle or skin. COMP plays diverse roles including the regulation of collagen secretion and fibrillogenesis, and the proliferation of chondrocytes in cartilage, the enhancement of cellular attachment and the inhibition of thrombin in blood vessels, the resistance to mechanical stress in tendons, the tissue remodeling in systemic sclerosis, or the trigger of the alternate pathway of the complement in the immune system [29,30,31]. In cartilage, COMP has shown promise as a diagnostic and prognostic indicator for OA. Serum COMP level is indeed elevated in OA patients, and could serve as a prognostic biomarker for the early diagnosis of this joint disease [32,33].

COMP protein is composed of five identical glycoprotein subunits (Figure 2). Each subunit consists of 757 amino acids that interacts close to their N-terminal extremity, resulting in a bouquet-like arrangement of the five monomers and forming a cavity in which different hydrophobic compounds like Vitamin D_3_ and all-*trans* retinol can reside [31,34]. This domain is followed by flexible regions of four EGF-like domains and eight TSP calmodulin-like repeats, which present numerous Ca^2+^ binding sites and are essential for correct protein folding [29]. The C-terminal globular domain binds to extracellular matrix proteins, namely collagens I, II and IX, aggrecan and fibronectin. This association of COMP with major components of the cartilage extracellular matrix supports a role of this protein in the structural integrity of cartilage. These bindings of COMP to extracellular matrix proteins appear to depend on divalent cations (Ca^2+^, Mg^2+^ or Mn^2+^) [31]. Additionally, COMP interacts also with cell surface proteins that influence various chondrocyte activities (cell adhesion, migration and intracellular communication), as well as with growth factors, such as Transforming Growth Factor beta or Bone Morphogenetic protein [31].

In humans, COMP is encoded by a 9,532 bp-long gene present in chromosome 19 at position 19p13.1 (GRCh38/hg38: chr19:18,782,773–18,791,305) and composed of 19 exons (Figure 2). To date, 148 unique public variants on COMP gene have been referenced in the Global Variome shared LOVD database (https://databases.lovd.nl/shared/genes/COMP, accessed on 2 April 2020), of which 131 are predicted to be pathogenic. Most of the variations correspond to substitutions (82%; 108/131) or deletions (12%; 16/131). The majority of *COMP* variations (>85%) are located in exons 9–14 which encoded the TSP type-3 repeat domains. These mutations compromise calcium binding and protein folding, leading to the retention of COMP in the chondrocyte endoplasmic reticulum. This causes oxidative and inflammation processes leading to chondrocyte death and loss of long bone growth [29]. These mutations are causal for the development of MED and pseudoachondroplasia (Table S1). These two autosomal dominant osteochondrodysplasias result in varying degrees of short stature, joint pain, joint laxity, joint stiffness and early-onset OA [29,35]. Only two *COMP* mutations are reported in OA patients without dysplasia, suggesting that predisposition to OA could correspond to the mild end of the *COMP* gene mutations spectrum [36,37].

In this report, we have identified a germline mutation (c.1358C>T, p.Asn453Ser) in the *COMP* gene in a family with an extreme phenotypic variability. The mutated residue is well conserved in different species, and present at one Ca^2+^ binding site in a TSP-type 3 repeat domain of COMP protein. Molecular dynamics simulations suggest that the mutation does not influence directly the Ca^2+^ bound in the target calcium binding site but it induces an important mobility in its environment and so destabilizes the COMP protein inducing the Ca^2+^ release from neighboring calcium binding sites. Therefore, along with the simulation results, the mutation will affect protein folding and cause a reduction in the number of bound calcium ions. This suggests that COMP function may be altered, since it has been previously described that mutations of coordination residues of others Ca^2+^ binding sites present in the same TSP-type 3 repeat domain of COMP protein disturb the local conformations of the type 3 repeats and reduced the binding to the extracellular matrix (collagens I, II and IX) [38,39].

The mutation is absent from gnomAD and was previously described once in a family with a severe form of MED [28]. Herein, the mutation was identified in three first-degree relatives (III.1, III.3 and III.4), which present three distinct phenotypes. The other family members were not screened for the mutation due to unavailability of genomic DNA. The index case (III.1) is affected by idiopathic early-onset hip OA without hip dysplasia. This was unexpected in view of the literature, since the mutation was associated with severe MED [28]. Contrariwise, one of her brothers (III.2) has MED and secondary OA requiring bilateral hip and knee prosthesis (from 22 to 35 years old). However, in this relative, we could not investigate the presence or not of the mutation. Another of her brothers (III.3), who also carries the mutation, was also diagnosed with MED, which is in accordance with the initial report from Briggs, and coherent with the phenotype induced by other COMP mutations. In addition, this family member (III.3) developed spontaneous osteonecrosis of the medial femoral condyle at 45 years old, while another brother (III.5) developed fractures of the femoral neck at 23 and 35 years old, suggesting some bone alterations in these individuals. This is coherent with a role of COMP in osteogenesis [40], and in pathological processes of non-traumatic osteonecrosis of the femoral head [41].

Unexpectedly, the sister (III.4) who was 43 at the date of recruitment, also carries the *COMP* mutation. She is affected by an atypical syndrome, characterized by dwarfism, mild facial dysmorphic features, brachydactyly without clubbing, moderate intellectual disability, a sensorineural hearing loss, a premature ovarian insufficiency and peripheral inflammatory arthritis of unknown cause (seronegative on complete auto-immune analyses), without OA nor dysplasia. Her phenotype does not correspond to MED nor pseudoachondroplasia. We suggest that at least two mutations could be involved in the atypical clinical presentation of this patient. Considering the role of *COMP* mutations in inflammation process, the Asn453Ser mutation would lead to inflammation responsible for peripheral inflammatory arthritis [29]. Another alteration would be causal for the new syndrome observed. In addition, we cannot exclude that the absence of OA is due to the younger age of the patient compared to the index case.

The non-diagnostic of hip dysplasia in the index case (III.1) and in her sister (III.4) might be explained by the difficulty to diagnose mild form of MED due to the specific expression of the disease in childhood, only characterized by a nonspecific expression of early-onset OA in adults [37]. We also suggest that modifier factors, such as other genetic alterations, epigenetic modifications or the environment, may play a role in the expression of this mutation, resulting in modification of the phenotypic severity of the disease.

## 5. Conclusions

In conclusion, this report extends the phenotypic spectrum associated with *COMP* mutations. In this study, the p.Asn453Ser COMP mutation was not linked to an inherited form of severe MED (contrary to what is described in the initial report from Briggs or observed with other mutations in *COMP* gene), but was associated with a marked intrafamilial variability. Individuals having the mutation present cartilage or bone alterations which induced different phenotypes: MED with secondary OA, but also primary OA, osteonecrosis, or peripheral inflammatory arthritis. Future investigations on modifier factors should be conducted to better understand the phenotypic heterogeneity and to improve the interpretation of *COMP* mutations in order to improve the medical care management of patients carrying mutations.

## Data Availability

The datasets used and/or analysed during the current study are available from the corresponding author on reasonable request.

## Supplementary Materials

The following is available online at https://www.mdpi.com/article/10.3390/biom11101460/s1, Table S1: COMP mutations involved in Multiple epiphyseal dysplasia (MED) and in Pseudoachondropla-sia (PSACH) according Uniprot databases (UniProtKB-P49747).
