# Supplementary material for: Description of Joint Alterations Observed in a Family Carrying p.Asn453Ser COMP Variant: Clinical Phenotypes, In Silico Prediction of Functional Impact on COMP Protein and Stability, and Review of the Literature
Source: Biomolecules. 2021 Oct 5;11(10):1460. doi: 10.3390/biom11101460 (PMC8533395; doi:10.3390/biom11101460)
Supplement: Supplementary file 1 [file biomolecules-11-01460-s001.zip › biomolecules-1364877-supplementary.pdf]

Supplementary Table S1. COMP mutations involved in Multiple epiphyseal dysplasia (MED) and in Pseudoachondroplasia (PSACH) according Uniprot databases (UniProtKB - P49747).

Swiss-Prot variant identifier	Position(s)	Amino-acid change	Disorder	References
**In EGF-like domain**				
VAR_066789	167	G -> E	MED	[1]
VAR_066790	234	P → S	PSACH	[1]
**In TSP type 3 repeat domain**				
VAR_026239	276	P → R	MED	[1], [2]
VAR_066791	290	D → G	PSACH	[1]
VAR_007614	290	D → N	PSACH; mild form	[3]
VAR_066792	298	S → L	MED (overlap with mild PSACH)	[1]
VAR_007615	299	G → R	PSACH	[1], [3]
VAR_066793	311	A → D	MED	[1]
VAR_066794	317	D → G	MED; atypical form	[1]
VAR_066795	326	D → G	MED	[1]
VAR_066796	326	D → Y	PSACH	[1], [3]
VAR_007616	328	C → R	PSACH; mild form	[4], [5]
VAR_066797	341 – 342	Missing	PSACH	[1]
VAR_007617	342	D → Y	MED; Fairbank type	[4], [5]
VAR_066798	348	C → F	MED	[1]
VAR_017102	348	C → R	PSACH	[6]
VAR_007618	349	D → V	PSACH; mild form	
VAR_066799	350 – 372	Missing	PSACH	[1]
VAR_007619	361	D → V	MED; Fairbank type	
VAR_007620	361	D → Y	MED	[5], [7]
VAR_007621	367 – 368	Missing	MED	[5]
VAR_007622	371	C → S	MED; Fairbank type	[1], [8]
VAR_066800	371	C → Y	MED	[1]
VAR_007623	372	Missing	PSACH	[5]
VAR_066801	374	D → N	MED	[1]
VAR_007624	374	Missing	PSACH; mild form	
VAR_066802	376	D → N	MED	[1]
VAR_066803	378	D → V	PSACH	[1]
VAR_066804	385	D → N	MED; atypical form	[1]
VAR_066805	385	D → Y	MED; atypical form	[1]
VAR_066806	385	Missing	MED	[1]
VAR_007625	387	C → G	PSACH; mild form	
VAR_066807	387	C → R	PSACH	[1]
VAR_066808	397	D → H	MED	[1]
VAR_007626	391 – 394	PNSD → V	PSACH	[5]
VAR_066809	402 – 404	GIG → VC	PSACH	[1]
VAR_066810	404	G → R	MED	[1]
VAR_007627	408	D → Y	MED	[5]
VAR_066811	410	C → Y	MED (overlap with mild PSACH)	[1]
VAR_066812	415	N → K	MED	[1]

VAR_026240	420	D → A	MED	[2]
VAR_066813	427	G → E	MED	[1]
VAR_066814	430 – 432	CDS → LWC	MED	[1]
VAR_007628	440	G → E	PSACH; mild form	
VAR_007629	440	G → R	PSACH	[1], [5], [8]
VAR_066815	446	D → N	PSACH	[1]
VAR_066816	448	C → S	PSACH	[1]
VAR_007630	453	N → S	MED; Fairbank type	[9]
VAR_066817	457	Missing	MED	[1]
VAR_007631	459	Missing	PSACH; severe form	[5], [10]
VAR_007632	468	C → Y	PSACH; severe form	[5], [10]
VAR_007633	469	Missing	PSACH; severe form	[5], [7], [11]
VAR_007634	472	D → Y	PSACH; severe form	[5], [10]
VAR_066818	473	D → DD	MED	[1]
VAR_007635	473	D → G	PSACH; severe form	
VAR_066819	473	D → H	PSACH	[1]
VAR_007636	473	Missing	PSACH; severe form.	[1]
VAR_066820	475	D → N	PSACH	[1]
VAR_007637	482	D → G	PSACH	[1], [8]
VAR_066821	501	G → D	MED	[1]
VAR_066822	507	D → G	PSACH	[1], [12]
VAR_066823	511	D → G	PSACH	[1]
VAR_007638	513 – 516	Missing	PSACH; mild form	[8]
VAR_066824	515	D → G	PSACH	[1]
VAR_007639	518	D → N	PSACH; mild form	
VAR_007640	523	N → K	MED; Ribbing type	[1], [13]
VAR_066825	529	T → I	PSACH	[1], [14]
**In TSP C term domain**				
VAR_007642	585	T → R	MED and PSACH	[1], [9]
VAR_007641	585	T → M	PSACH; mild form and MED1	[1], [2]
VAR_066826	718	R → P	MED	[1]
VAR_066827	718	R → W	MED	[1], [15]
VAR_017103	719	G → D	PSACH; severe	[16]
VAR_066828	719	G → S	PSACH	[1]

References:

[1] G. C. Jackson et al., « Pseudoachondroplasia and Multiple Epiphyseal Dysplasia: A 7-Year Comprehensive Analysis of the Known Disease Genes Identify Novel and Recurrent Mutations and Provides an Accurate Assessment of Their Relative Contribution », Hum. Mutat., vol. 33, no 1, p. 144-157, janv. 2012, doi: 10.1002/humu.21611.

[2] M. Czarny-Ratajczak et al., « A Mutation in COL9A1 Causes Multiple Epiphyseal Dysplasia: Further Evidence for Locus Heterogeneity », Am. J. Hum. Genet., vol. 69, no 5, p. 969-980, nov. 2001.

[3] S. Ikegawa et al., « Novel and recurrent COMP (cartilage oligomeric matrix protein) mutations in pseudoachondroplasia and multiple epiphyseal dysplasia », Hum. Genet., vol. 103, no 6, p. 633-638, déc. 1998, doi: 10.1007/s004390050883.

[4] M. D. Briggs et al., « Pseudoachondroplasia and multiple epiphyseal dysplasia due to mutations in the cartilage oligomeric matrix protein gene », Nat. Genet., vol. 10, no 3, p. 330-336, juill. 1995, doi: 10.1038/ng0795-330.

[5] J. Loughlin et al., « Identification of five novel mutations in cartilage oligomeric matrix protein gene in pseudoachondroplasia and multiple epiphyseal dysplasia », Hum. Mutat., vol. 11, no S1, p. S10-S17, 1998, doi: 10.1002/humu.1380110105.

[6] S. Unger, J. Korkko, D. Krakow, R. S. Lachman, D. L. Rimoin, et D. H. Cohn, « Double heterozygosity for pseudoachondroplasia and spondyloepiphyseal dysplasia congenita », Am. J. Med. Genet., vol. 104, no 2, p. 140-146, 2001, doi: 10.1002/ajmg.10062.

[7] J. Thur et al., « Mutations in Cartilage Oligomeric Matrix Protein Causing Pseudoachondroplasia and Multiple Epiphyseal Dysplasia Affect Binding of Calcium and Collagen I, II, and IX », J. Biol. Chem., vol. 276, no 9, p. 6083-6092, mars 2001, doi: 10.1074/jbc.M009512200.

[8] S. Susie, J. McGrory, J. Ahier, et W. G. Cole, « Multiple epiphyseal dysplasia and pseudoachondroplasia due to novel mutations in the calmodulin-like repeats of cartilage oligomeric matrix protein », Clin. Genet., vol. 51, no 4, p. 219-224, 1997, doi: 10.1111/j.1399-0004.1997.tb02458.x.

[9] M. D. Briggs et al., « Diverse mutations in the gene for cartilage oligomeric matrix protein in the pseudoachondroplasia-multiple epiphyseal dysplasia disease spectrum », Am. J. Hum. Genet., vol. 62, no 2, p. 311-319, févr. 1998, doi: 10.1086/301713.

[10] J. T. Hecht et al., « Mutations in exon 17B of cartilage oligomeric matrix protein (COMP) cause pseudoachondroplasia », Nat. Genet., vol. 10, no 3, p. 325-329, juill. 1995, doi: 10.1038/ng0795-325.

[11] H. Chen, M. Deere, J. T. Hecht, et J. Lawler, « Cartilage Oligomeric Matrix Protein Is a Calcium-binding Protein, and a Mutation in Its Type 3 Repeats Causes Conformational Changes », J. Biol. Chem., vol. 275, no 34, p. 26538-26544, août 2000, doi: 10.1074/jbc.M909780199.

[12] M. Deere, T. Sanford, H. L. Ferguson, K. Daniels, et J. T. Hecht, « Identification of twelve mutations in cartilage oligomeric matrix protein (COMP) in patients with pseudoachondroplasia », Am. J. Med. Genet., vol. 80, no 5, p. 510-513, 1998, doi: 10.1002/(SICI)1096-8628(19981228)80:5<510::AID-AJMG14>3.0.CO;2-F.

[13] R. Ballo, M. D. Briggs, D. H. Cohn, R. G. Knowlton, P. H. Beighton, et R. S. Ramesar, « Multiple epiphyseal dysplasia, ribbing type: A novel point mutation in the COMP gene in a South African family », Am. J. Med. Genet., vol. 68, no 4, p. 396-400, 1997, doi: 10.1002/(SICI)1096-8628(19970211)68:4<396::AID-AJMG4>3.0.CO;2-K.

[14] J. Kennedy et al., « COMP mutation screening as an aid for the clinical diagnosis and counselling of patients with a suspected diagnosis of pseudoachondroplasia or multiple epiphyseal dysplasia », Eur. J. Hum. Genet. EJHG, vol. 13, no 5, p. 547-555, mai 2005, doi: 10.1038/sj.ejhg.5201374.

[15] J. Kennedy et al., « Novel and recurrent mutations in the C-terminal domain of COMP cluster in two distinct regions and result in a spectrum of phenotypes within the pseudoachondroplasia – multiple epiphyseal dysplasia disease group », Hum. Mutat., vol. 25, no 6, p. 593-594, 2005, doi: 10.1002/humu.9342.

[16] A. Mabuchi et al., « Novel mutation in exon 18 of the cartilage oligomeric matrix protein gene causes a severe pseudoachondroplasia », Am. J. Med. Genet., vol. 104, no 2, p. 135-139, 2001, doi: 10.1002/ajmg.10067.
